# Bone and Cartilage Conduction—Volume II

**DOI:** 10.3390/audiolres15040093

**Published:** 2025-08-01

**Authors:** Tadashi Nishimura, Takanori Nishiyama

**Affiliations:** 1 Department of Otolaryngology-Head & Neck Surgery, Nara Medical University, Nara 634-8521, Japan; 2 Department of Otorhinolaryngology-Head and Neck Surgery, Keio University School of Medicine, Tokyo 108-8345, Japan

Air conduction is the primary pathway for hearing sounds and is widely utilized in various hearing devices. In contrast, other forms of sound conduction—such as bone and cartilage conduction—have not been as commonly applied. However, recent advancements in device development have expanded the applications of these alternative conduction methods across various fields.

In the previous Special Issue on “Bone and Cartilage Conduction”, numerous basic and clinical studies on bone and cartilage conduction were published [1,2], reaffirming the growing interest among researchers in this area. To further explore the potential and promote the application of both bone and cartilage conduction, this Special Issue similarly addresses the mechanisms and practical uses of these methods.

Since 2017, a new hearing device utilizing cartilage conduction has been developed and released in Japan. It has gained rapid popularity and is now recognized as a major type of hearing device. Reflecting this trend, most of the contributions in this issue focus on cartilage conduction and hearing aids, with studies reported from various institutions in Japan. We believe that this content will attract considerable interest among researchers—not only in Japan, where such devices are already available, but also in countries where cartilage conduction hearing aids have yet to be introduced. We hope this issue will contribute to a deeper understanding of the field and encourage further international research and development.

This issue includes one review concerning cartilage conduction hearing aids (contribution 1), five research articles (contributions 2–6), one brief report (contribution 7), and one case report (contribution 8). All the research articles focus on cartilage conduction: two of them present basic research, and the other three studies address clinical applications of cartilage conduction hearing aids that are currently in use. The basic research papers report on the (contribution 2), and on studies of sound transmission pathways (contribution 3). The remaining three research articles and the brief report examine cartilage conduction hearing aids, discussing suitable candidates and the effectiveness of the devices (contributions 4–7). The case report explores the effects of using a bone conduction hearing aid as a vibratory stimulus (contribution 8). This study is of particular interest, demonstrating how sensory substitution can deliver sound to patients for whom cochlear implants are not feasible.

This scientific collection is expected to be of interest to a range of professionals, including audiologists, otolaryngologists, physiologists, and acoustic engineers.
